# Plankton active response to turbulence enables efficient transport

**DOI:** 10.1242/jeb.251123

**Published:** 2025-12-19

**Authors:** Michelle H. DiBenedetto, Rémi Monthiller, Christophe Eloy, Lauren S. Mullineaux

**Affiliations:** ^1^ Department of Mechanical and Aerospace Engineering, Princeton University, Princeton, NJ 08544, USA; ^2^ Aix-Marseille University, CNRS, Centrale Med, IRPHE, 13384 Marseille, France; ^3^ Department of Biology, Woods Hole Oceanographic Institution, Woods Hole, MA 02543, USA

**Keywords:** Larval swimming, Veliger, Behavior, Hydrodynamics

## Abstract

Although plankton typically have slow swim speeds relative to ocean flows, they can potentially enhance their transport by exploiting certain flow features. For example, a theorized ‘surfing’ strategy describes how plankton can preferentially sample upwelling areas of the flow by simply sensing and actively reorienting in response to local velocity gradients. In this study, we present the first experimental evidence that real plankton may be able to surf turbulence. We studied the bottom-heavy, planktonic larval snail *Crepidula fornicata* as our model organism. By observing these plankton in a jet-stirred turbulence tank, we show that they indeed have complex responses to velocity gradients. In particular, we found that they actively rotate to oppose the local vorticity, which contrasts with the typical passive, gyrotactic response. We compared our observations with those of simulated surfing plankton to demonstrate the applicability of the surfing theory to our data, where we found good agreement. Finally, we observed that the real plankton can preferentially sample upwelling areas of the flow in some cases, enhancing their transport relative to their swimming speed alone, similar to the proposed surfing theory.

## Teaser

**Highlighted Article:** Plankton actively tilt against flow rotation, exploiting turbulent flow structures to enhance their upward transport beyond their swimming capabilities alone.

## INTRODUCTION

The plankton in the ocean consist of a wide variety of organisms from bacteria to jellyfish. And while plankton, by definition, are species that predominantly drift along with the currents, this does not mean that assuming plankton ‘go with the flow’ is an accurate representation of their behavior in a turbulent ocean (Wheeler et al., 2019). Plankton respond to the flow around them both actively and passively in ways that can alter their net transport. Ecologically, understanding planktonic vertical transport is important because it modulates the plankton's position in the water column, controlling their exposure to currents, nutrients and sunlight. Water column position is in part mediated by plankton swimming; however, plankton swimming speeds are often much slower than the mean currents or characteristic fluctuating velocities near the ocean surface (Franks et al., 2022). And yet, their net speed may in fact differ as a result of their interactions with turbulence (e.g. Fuchs et al., 2013; Michalec et al., 2015; DiBenedetto et al., 2022). Of interest is a recently theorized ‘surfing strategy’, which describes how swimming plankton could effectively double their net speed in turbulence compared with still water by preferentially sampling the fluid velocity fluctuations (Monthiller et al., 2022). However, it is still unclear whether real plankton exhibit this behavior.

It is well established that small changes to planktonic behavior can have large-scale effects on their transport. One well-studied passive behavior in plankton is bottom heaviness, i.e. gyrotaxis in a viscous fluid (Kessler, 1985). Gyrotactic swimmers have been of interest because of the array of behaviors they exhibit in different flows. Gyrotactic trapping was described by Durham et al. (2009), where gyrotactic swimmers can form horizontal thin layers under steady horizontal shear flow. In isotropic turbulence, gyrotactic swimmers can ‘unmix’ into patches (Durham et al., 2013), and can preferentially sample different parts of the turbulence depending on their shape and relative swimming speed. Preferential sampling in this case refers to when plankton oversample certain turbulence statistics, on average, relative to a fluid tracer which faithfully follows the flow. For example, in homogeneous turbulence with no mean flow, a tracer would also experience no mean flow. However, a plankter (or particle) which preferentially samples the turbulent fluctuations could experience a net flow. While previous work has shown that spherical gyrotactic swimmers will preferentially sample downwelling areas of the flow and thereby reduce their upward transport (Zhan et al., 2014; Gustavsson et al., 2016), elongated, non-spherical swimmers can preferentially sample upwelling areas of the flow to enhance their upward transport (Borgnino et al., 2018; Lovecchio et al., 2019). These studies show how even simple behavioral models, such as a swimmer with a constant swim speed and a passive gyrotactic torque, can have meaningfully altered transport when studied in a turbulent flow. Many small, motile phytoplankton with small Reynolds numbers (*Re*<<1) have been shown to appropriately behave as simple gyrotactic swimmers (Durham et al., 2009, 2013; Zeng et al., 2022); however it is not clear that these dynamics apply to other types of plankton with more complex behavior.

Zooplankton are typically larger than phytoplankton and have more diverse morphologies and swimming behaviors. Therefore, phytoplankton models of behavior cannot directly be mapped onto larger zooplankton which have intermediate Reynolds numbers. While many planktonic larvae are also bottom heavy and thus have passive gravitational stability (Strathmann and Grünbaum, 2006; Clay and Grünbaum, 2010; Wheeler et al., 2016), it is not clear that gyrotaxis adequately describes all of their orientational behavior, even though the gyrotactic model has been previously applied to some planktonic larvae (Fuchs et al., 2013, 2015a,b). The deviation from a passive model of orientation, i.e. gyrotaxis, is in part due to the fact that many plankton can also actively control their orientation. For example, many larvae exhibit helical swimming behaviors with varied angular velocities (e.g. Chan and Grünbaum, 2010; Bearon, 2013; Maciejewski et al., 2019; Krishnamurthy et al., 2020), or more complex swimming patterns that cannot be categorized as helical swimming (DiBenedetto et al., 2021). However, studies have yet to fully elucidate how both the active and passive orientational behavior by larvae modulate their transport in turbulence.

This study builds on previous work we conducted studying the swimming response of zooplankton to turbulence, in which we observed *Crepidula fornicata* larvae swimming in a jet-stirred turbulence tank (DiBenedetto et al., 2022). Overall, we found that these plankton do increase their upward swimming speed in response to turbulence, and that their response is best correlated to the local fluid acceleration. However, we also found that the plankton modulated their response based on the average background level of turbulence, suggesting that they are able to integrate flow information over time to influence their behavior. In contrast, in this study, we focused on orientational behavioral responses to turbulence, studying how the plankton both actively and passively respond to the flow around them. Because we cannot explicitly distinguish between these two behaviors in our data, we compared our results with simulations of plankton with two different behavioral models in order to interpret the observations. We examined models of both passive gyrotaxis and active surfing, the former of which theoretically results in a slowdown of upward swimming (spherical) plankton in turbulence, and the latter of which results in a speeding up of the same plankton in turbulence. Overall, we found that the behavior of the real plankton in this study tends to agree more with that of surfing than gyrotactic plankton.

Below, we first describe the methodology, including both the laboratory experiments and the simulations. Next, we review the gyrotaxis and surfing models of behavior. Finally, we report and discuss the results of our study.

## MATERIALS AND METHODS

### Laboratory experiments

**Fig. 1.**
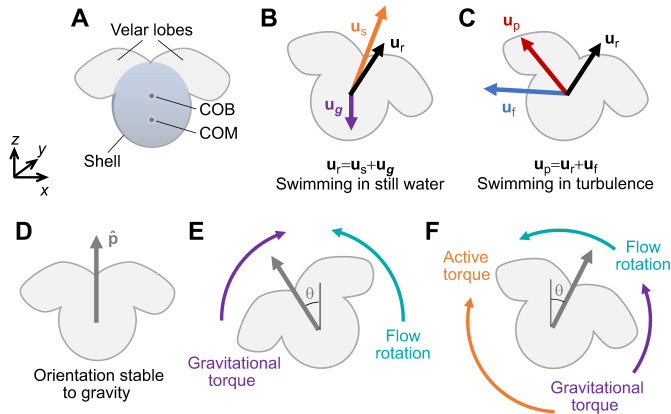
**Plankton diagram and kinematic schematics.** (A) Diagram of veliger larvae of *Crepidula fornicata* (not drawn to scale) with representative relative locations of center of buoyancy (COB) and center of mass (COM). (B) In still water, plankton relative velocity **u**_r_ is the sum of plankton swimming velocity **u**_s_ and gravitational sinking velocity **u*_g_***. The plankton is assumed to swim in the direction it is oriented, as denoted by the unit vector 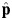 (see D). (C) The total plankton velocity in turbulence can be divided into the sum of the plankton relative velocity **u**_r_ and fluid velocity interpolated onto the plankter **u**_f_. (D) A larva drawn in its stable upright posture. (E) Gyrotactic behavior with passive stabilization resulting in tilting in the direction of flow rotation. (F) Conceptual model for active stabilization behavior resulting in tilting opposing flow rotation consistent with surfing.

We use the veliger larval stage of the slipper snail *Crepidula fornicata* as our model organism. These plankton are approximately spherical with a heavy shell. They have a ciliated velum divided into two velar lobes which they use to swim that sits above their shell in their upright posture, as shown in Fig. 1A. Their cilia create a current used for both feeding and propulsion. In general, their swimming speed smoothly varies, and they have a strong preference for swimming up due to their bottom heaviness (Chan et al., 2013; DiBenedetto et al., 2021). Their bias for swimming up is also related to the fact that they are negatively buoyant, so they need to create upward propulsion in order not to sink. The diameter of the larvae range from about 0.3 to 1 mm and they can swim at speeds of 1–4 mm s^−1^ in still water (Chan et al., 2013), which puts them in an intermediate Reynolds number regime (of order unity) defined as *Re*_p_=*V*_swim_ *d*/ν=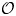
(1), where *V*_swim_ is their swim speed, *d* is their diameter and ν≈10^−6^ m^2^ s^−1^ is the kinematic viscosity of sea water. This preference for swimming up, their active orientational behavior, spherical shape and intermediate Reynolds number are what makes them an appropriate model organism for this study. We expect them to be able to respond to the flow, but to not vary their swimming speed too much, when, for example, compared with copepods, which exhibit highly unsteady and irregular jump responses in turbulence (Kiørboe and Visser, 1999; Michalec et al., 2015).

We conducted controlled laboratory experiments to observe the effects of turbulence on plankton swimming behaviors. Two sets of larvae were used in the experiments: an ‘early stage’ and a ‘late stage’. The former were 2 days old, with an average diameter of 488 μm, and the latter were 12 days old, with an average diameter of 758 μm. The larvae were put in a 30.5 cm cubic jet-stirred turbulence tank designed to create low Reynolds number isotropic turbulence. These data come from a previously published study (DiBenedetto et al., 2022), where more details can be found on plankton collection and rearing, the turbulence tank and the experimental procedure. To summarize, experiments were run at randomized turbulence levels: ‘low’, ‘medium’ and ‘high’. Average flow statistics are reported for each case in Table 1. One common way to characterize turbulence intensity is via the dissipation rate ε. The levels in our experiments ranged from values of the order of 10^−3^ to 10^−2^ cm^−2^ s^−3^. This range corresponds to observations in the upper 20 m of the continental shelf, estuaries and tidal channels (Fuchs and Gerbi, 2016), and the upper 10 m of the open ocean (Franks et al., 2022). We used particle image velocimetry (PIV) to measure the flow field in the center plane of the tank and used particle tracking to measure the velocity of the plankton that intersected the 2D plane. All measurements were taken in the *x*–*z* plane, corresponding to *u* and *w* velocities, and therefore only the out-of-plane vorticity component ω*_y_* was measured. The image field-of-view was approximately 5.4 cm×5.4 cm with 377 pixels cm^−1^ resolution. PIV processing was done with LaVision software and the resultant PIV vectors were resolved to 32×32 pixel boxes with 25% overlap.

**Table 1.** Experimental overview

	Turbulence level			
	None	Low	Medium	High
*Re*_λ_	–	27	52	65
η (cm)	–	0.14	0.11	0.082
ε (cm^2^ s^−3^)	–	0.0025	0.0081	0.022
Early stage ⟨*w*_r_⟩ (mm s^−1^)	0.69±0.02	0.037±0.018	0.48±0.023	1.4±0.22
Late stage ⟨*w*_r_⟩ (mm s^−1^)	0.24±0.03	−0.046±0.031	0.58±0.031	0.37±0.030

The table lists the average Reynolds number *Re*_λ_, Kolmogorov length scale η and dissipation rate ε for the three turbulence levels and still water. Also included is the average vertical relative velocity of the larvae ⟨*w*_r_⟩ in each condition with 95% confidence intervals.

### Laboratory data analysis

In still water, the plankton velocity is due to the effects of swimming and gravitational sinking, which depend on the forces from propulsion, drag, buoyancy and gravity. This means that the larval relative velocity **u**_r_ can be approximated as the sum of a swimming velocity **u**_s_ and terminal settling velocity **u*_g_***, as depicted in Fig. 1B. We label the swimming speed (the magnitude of **u**_s_) as *V*_swim_. We emphasize that there is inherent uncertainty in estimating **u*_g_***, which is a function of the mass, volume and drag coefficient of the larvae. The mass and volume have variability across individuals, and the drag coefficient depends on velar posture, which is variable and depends on the larval swimming behavior. Therefore, we did not attempt to quantify **u*_g_***, and instead we analyzed **u**_r_ as a proxy for swimming. Note that the horizontal component of **u**_r_ is entirely due to swimming. In turbulence, the larval velocity is additionally affected by the background flow. In this case, we measured the total larval velocity **u**_p_, which is the sum of **u**_r_ and the fluid velocity **u**_f_, which represents the flow field that the plankton are experiencing (see Fig. 1C). By interpolating the PIV field around the plankton onto the centroids of the plankton (*x*_p_,*z*_p_), we were able to estimate **u**_f_. We interpolated the fluid velocity and spatial gradients using a least-squares optimization of a 2D, second-order Taylor series function of the PIV data in an annulus greater than 0.27 cm and less than 0.53 cm away from the larval centroids (100 and 200 pixels). A sensitivity analysis showed that our results were not very sensitive to this choice. We calculated the plankton relative velocity **u**_r_ by taking the total plankton velocity **u**_p_ measured via particle tracking and subtracting the interpolated fluid velocity **u**_f_:
(1)

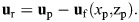
Note that we define the vector **u**_r_=(*u*_r_,*w*_r_) in 2D where *u*_r_ is the horizontal component in the *x*-direction and *w*_r_ is the vertical component in the *z*-direction.

In our experiments, the relative velocities of the larvae were on average larger in the vertical direction than in the horizontal direction because of their preference for swimming up. We report the average vertical relative velocity for each larval stage and each turbulence level in Table 1. Note that there is some response to the turbulence level: in our earlier study, we found that the plankton tend to increase their upward swim speed under increasing turbulence intensity and increasing local fluid acceleration (DiBenedetto et al., 2022).

Whereas in our previous study we focused on plankton vertical swimming behavior (DiBenedetto et al., 2022), in this study we instead focused on the orientational behavior of the plankton relative to the local flow gradients. Because the plankton are approximately spherical, measuring their orientation in the experimental images was not possible. As a proxy for orientation, we can consider the horizontal relative velocity *u*_r_; the larvae are typically observed in their upright position, and therefore their orientation should correlate to their horizontal swimming velocity, which is estimated via their horizontal relative velocity. We chose to avoid using a model to infer plankton kinematics, which, for example, could be done either by using a Maxey–Riley model to estimate the forces and torques on the plankter (e.g. Fuchs et al., 2015a,b) or by estimating swimming by subtracting an assumed sinking speed from the measured relative velocity. We chose this approach to avoid uncertainty in estimating plankton drag coefficients and mass distribution, which would be needed to estimate the forces and the sinking speed of the larvae. As previously noted, the drag coefficient of these plankton is not constant because they manipulate their velum to adjust their swimming velocity (Chan et al., 2013). Finally, to further constrain our analysis, we only considered the relative horizontal velocity *u*_r_ when the larvae are swimming up, to avoid circumstances when the plankton are either upside down or sinking.

### Numerical simulations

To model a homogeneous isotropic turbulent flow, we used the pseudo-spectral, open-source solver *Snoopy* (Lesur and Longaretti, 2005, 2007). The Navier–Stokes equations were solved for an incompressible fluid with kinematic viscosity ν=0.004 (arbitrary units), in a tri-periodic domain of size *l*=1 (arbitrary units) with resolution of *n*×*n*×*n* with *n*=128. We used an external forcing delta-correlated in time and localized in spectral space (3/2<|*k*|*l*<5/2, where *k* is the wave vector) to make the flows statistically steady. The simulations used in this study come from Monthiller (2022), where more details can be found. We chose a simulation with *Re*_λ_=11 because although this Reynolds number is somewhat lower than that in the experiments, Monthiller (2022) showed their results to be qualitatively similar over a wide range of Reynolds numbers.

We used the root mean square velocity *u*_rms_ and the integral length scale *L*, computed as follows:
(2)

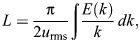
where *E* is the spectral energy density, to characterize the simulated turbulent flows. The parameters of the homogeneous isotropic turbulence simulations performed with *Snoopy* are summarized as follows: *Re*_λ_=11, *L*/η=35, *T_L_*/τ_η_=19, *u*_rms_/*u*_η_=1.8, where η, τ_η_ and *u*_η_ characterize the Kolmogorov scales and *T_L_*=*L*/*u*_rms_ is the integral time scale. The Lagrangian equations of plankter motion were integrated using the open-source code SHELD0N (Monthiller et al., 2022).

### Conceptual plankton model

We use an idealized framework to describe plankton swimming in turbulence to compare with and contextualize our laboratory observations. In this framework, we assume plankton are smaller than or of the same order as the Kolmogorov length scale η. This means that the flow gradients are uniform across the plankter and that the flow they experience can be described by the velocity at their centroid **x**_p_(*t*). We further assume the plankton do not affect the flow around them, and that they are advected by the fluid velocity **u**_f_ plus a constant swimming speed *V*_swim_. Their swimming direction is denoted by the unit vector 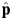:
(3)

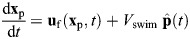
Although we have assumed *V*_swim_ is constant in this case in order to focus on orientational dynamics, in general both the plankton we consider in this study and others can vary their swim speed (e.g. Chan et al., 2013; DiBenedetto et al., 2021; Visser and Kiørboe, 2006; True et al., 2020). This description of plankton swimming allows us to directly compare two models of plankton orientation: passive gyrotaxis and active surfing. In reality, we expect real plankton to have both a passive and active orientational response, and we cannot precisely separate the two responses in our experimental observations.

### Gyrotaxis

Gyrotactic swimmers passively reorient based on the local fluid vorticity and their gyrotactic reorientation time scale *B* (Kessler, 1985). This reorientation time scale characterizes the restoring torque due to plankton bottom heaviness in a viscous fluid. The torque is in part controlled by how offset the plankton's center of mass is from their center of buoyancy. We write the equation of motion for their orientation following Pedley and Kessler (1987):
(4)

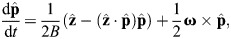
where 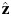 is the upward vertical unit vector which denotes their stable upright orientation due to gravity, and **ω** is the fluid vorticity evaluated at the plankter's centroid. This model is appropriate for spherical plankton, which is a good approximation of the plankton considered in our experiments. If the plankton were non-spherical, there would be an additional term that involves the aspect ratio of the plankton and the rate of strain tensor of the flow (Jeffery, 1922; Zhan et al., 2014; Gustavsson et al., 2016), which is most important for elongated plankton with high aspect ratio. Eqn 4 gives a fixed-point orientation determined by the vorticity and reorientation time scale: in two dimensions, we can define an orientation angle θ that is the angle between 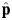 and the vertical direction. In this case, θ is then given by the equation sinθ=*B*ω for *B*ω<1. (In three dimensions, the equation is more complicated; see Eloy, 2024.) We see clearly from this expression that stronger vorticity results in a larger rotation.

Under this passive behavioral model, we expect vorticity to be correlated with the orientation of the plankton. We expect plankton to tilt with the vorticity, as is depicted in Fig. 1E.

### Surfing

Surfing describes an active orientational control strategy by plankton in response to the local flow gradients. The plankton's orientation is chosen to maximize their transport in a target direction, taken here to be the upward vertical. This is a strategy that has only been described theoretically and was derived in order to optimize plankton navigation with minimal sensing of the flow (Monthiller et al., 2022). Under the assumption that the plankton can reorient instantaneously, we can write an equation for their orientation as a function of time as follows:
(5)

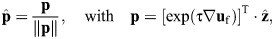
where τ is a constant free parameter which we refer to as the surfing time horizon, exp(·) is the matrix exponential and [·]^T^ is the transpose. Using simulations, we have previously shown that there exists an optimal τ for a given flow and *V*_swim_ value (Monthiller et al., 2022). Initial results from simulations of surfers in turbulence over a range of Reynolds numbers and *V*_swim_ values indicate that the optimal τ is of the order of the Kolmogorov time scale τ_η_ and that increasing swimming velocity tends to reduce the optimal τ (Monthiller, 2022). In the surfer simulations used in this study, we found the optimal τ=2τ_η_.

Under this active behavioral model, we expect vorticity to be anti-correlated with the orientation of the plankton. We expect plankton to tilt into the vorticity, as is depicted in Fig. 1F.

## RESULTS

### Plankton actively oppose fluid vorticity

**Fig. 2.**
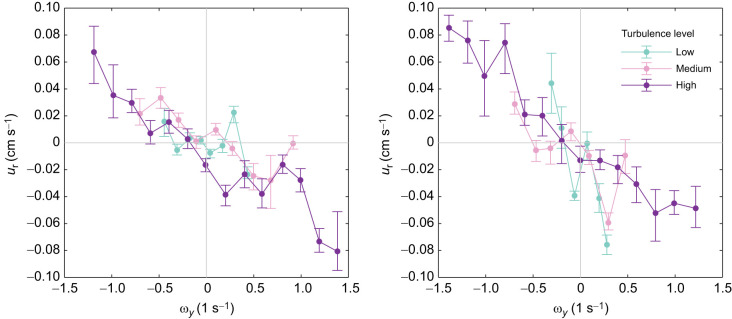
**Larval response to instantaneous vorticity.** Horizontal relative velocity *u*_r_ for larvae swimming up is binned by vorticity ω*_y_*, and plotted by background turbulence level. Early-stage larvae (left) and late-stage larvae (right) data are plotted separately. Error bars on bins denote 95% confidence intervals found by bootstrapping the data. Bins are equally spaced in vorticity for each turbulence level (with a spacing of 0.125 s^−1^ for the low turbulence level and 0.2 s^−1^ for the medium and high levels); only bins that have a minimum of 75 data points are shown. Corresponding correlation coefficients are reported in Table 2.

**Fig. 3.**
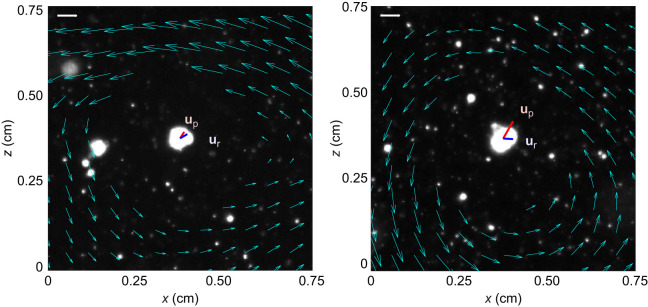
**Observations of larvae in turbulence in high vorticity regions, which corresponds to the conceptual model in Fig. 1F.** Early-stage larva (left) and late-stage larva (right) snapshots shown under high turbulence forcing. The vector field denotes fluid flow relative to the larval velocity vector: **u**_f_−**u**_p_. Larval velocity **u**_p_ and larval relative velocity **u**_r_ are plotted from the centroid of each larva. Scale bar: 5 mm s^−1^. Images were taken from raw experimental footage.

The main result from our experimental observations indicates a strong anti-correlation between the larval horizontal relative velocity and the fluid vorticity, consistent with surfing behavior. We observed this effect in both the early- and late-stage larvae and in all levels of turbulence, as seen in Fig. 2, where horizontal plankton relative velocity *u*_r_ was binned and plotted against vorticity ω*_y_* for each dataset. In this coordinate system, which is the same as that depicted in Fig. 3, negative vorticity (counterclockwise, out of the page) would tend to cause a gyrotactic plankter to swim in the negative *x* direction (to the left), and positive vorticity (clockwise, into the page) would tend to cause a gyrotactic plankter to swim in the positive *x* direction (to the right). Instead, we observed anti-gyrotactic behavior, and found that the larvae were on average swimming opposite to the expected tilt direction by vorticity. There was some variation in the slope of the relationships across turbulence levels, but they all tended to overlap within the uncertainty bounds.

**Table 2.** Pearson correlation coefficient *r* for horizontal relative velocity *u*_r_ correlated against vorticity ω*_y_* as plotted in Fig. 2

Larval stage	Turbulence level		
	Low	Medium	High
Early	*N*=5036, *r*=−0.10	*N*=3995, *r*=−0.18	*N*=6791, *r*=−0.26
Late	*N*=1243, *r*=−0.41	*N*=2102, *r*=−0.38	*N*=3675, *r*=−0.38

All *P*<0.001 (two-sided test of *H*_0_: *r*=0).

To demonstrate this effect, Fig. 3 shows snapshots of individuals tilting against the vorticity at the high turbulence level. The flow relative to the larva in both cases has strong negative ω*_y_* (counterclockwise), which would tend to tilt the bottom-heavy larvae to the left. However, in both cases, the larvae were observed swimming to the right with positive *u*_r_ values, demonstrating anti-gyrotactic behavior.

This result is further illustrated with a conceptual model in Fig. 1D–F. The larva in its stable, upright posture is shown for reference in Fig. 1D, and a larva tilting against the flow rotation is depicted in Fig. 1F. Because the larva is bottom heavy, when tilted to the right, a gravitational torque exists that would tend to rotate the larva counterclockwise (as drawn). In addition, the flow's vorticity also induces a counterclockwise torque on the larva. If the system is in equilibrium, then the larva will have to actively impose a torque to rotate clockwise, opposing the flow and gravitational torques. This suggests that we are observing an active orientational behavior, as the larval orientation cannot be fully explained without it.

We further assessed this observed correlation by calculating the Pearson correlation coefficient *r* between ω*_y_* and *u*_r_, which we report in Table 2 for each scenario. We found that all scenarios have statistically significant negative correlations, which agrees with the binned data plot in Fig. 2. However, the correlation coefficients are low for the early-stage larvae, where *r* ranges from −0.1 to −0.26, indicating high variability in the data. The late-stage larvae show stronger correlations, where *r* ranges from −0.38 to −0.41. This suggests that the late-stage larvae show a more consistent anti-gyrotactic response when compared with the early-stage larvae. And yet, the *r* values in all cases are rather modest, which is to be expected due to both the inherent variability in studying living organisms and the fact that the larvae are responding to a three-dimensional flow but we are only able to measure the flow and their behavior in two dimensions. Thus, this anti-gyrotatic orientational correlation is only observed in the average sense. Overall, we found that the larvae are responding actively to the flow in a way that could promote surfing, and we would expect the late-stage larvae to be better surfers than the early-stage larvae given their stronger response to the vorticity.

### Simulated surfers actively orient to oppose fluid vorticity

We next present results from the simulations of plankton in turbulence using the two different models of swimming behavior: passive gyrotaxis and active surfing (as described in Materials and Methods). To compare between the simulations and experiments, we non-dimensionalized the data. We normalized the fluid vorticity using the Kolmogorov time scale τ_η_=√(ν/ε). In the experimental data, we normalized *u*_r_ with the maximum vertical swimming velocity of the larvae observed in still water 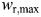 as a proxy for orientation. The observed maximum 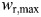 values were 0.18 and 0.33 cm s^−1^ for the early- and late-stage larvae respectively (as reported in DiBenedetto et al., 2022). In the simulations, we had direct access to the plankton orientation, so we plotted their orientation projected onto one horizontal axis *p_x_*=*u*_r_/*V*_swim_.

**Fig. 4.**
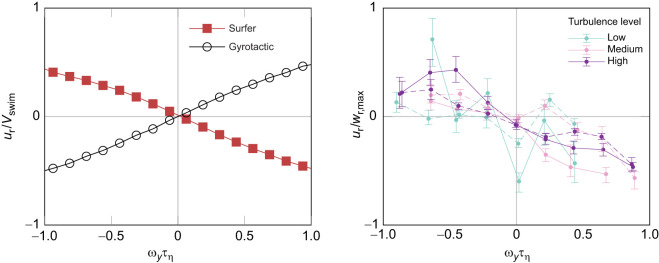
**Comparison of the response to instantaneous vorticity between simulations and experiments.** Both plots show normalized horizontal relative velocity 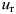 plotted against normalized out-of-plane vorticity 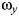. Left: simulation evaluated for a swimmer of swimming velocity *V*_swim_=*u*_η_ where *u*_η_ is the Kolmogorov velocity scale given by *u*_η_=(νε)^1/4^. The surfer has been provided its optimal time horizon τ=2τ_η_ and the gyrotactic swimmer is evaluated with a reorientation time scale *B*=τ_η_. Right: data from Fig. 2 renormalized for comparison with simulations. The still water maximum *w*_r_ values are used for normalizing the data. Early- and late-stage larvae are indicated by dashed and solid lines, respectively.

The results from the simulation are plotted in the left panel of Fig. 4, where it can be seen that the simulated surfer orientation indeed also shows a strong anti-correlation with vorticity, resembling the trend observed in experiments plotted in the right panel of Fig. 4. When comparing to the gyrotactic plankton orientation, which is also plotted in Fig. 4 (left), the opposite orientational behavior can be seen, as expected.

These simulations again support the result that the larvae in the experiment were not exhibiting traditional passive gyrotaxis, and instead were actively rotating to oppose the vorticity. The simulations plotted were from surfers with the optimal time horizon τ, which is the free parameter in the surfing model and describes how surfing plankton should weight their response to the velocity gradient. The similarity in the plots also indicates that the larval orientational behavior response to vorticity has good agreement with that of optimal surfers, i.e. surfers with the optimal time horizon.

### Plankton preferentially sample the flow

While we have shown how the orientational behaviors of the larvae resemble surfing behaviors, to truly confirm whether the larvae are surfing, we need to assess whether they preferentially sample the flow. In other words, we need to measure the average fluid vertical velocity experienced by the larvae relative to a fluid tracer. Measuring the effect of preferential sampling is not trivial in a closed, jet-stirred turbulence tank; this is because even though the tank was designed to have zero mean flow, small mean flows can arise in each experimental trial that are of the order of the velocities of interest. To account for this, we can consider each experimental trial and compare the average fluid velocity sampled by the larvae with the average fluid velocity in the tank in each trial. To be consistent and avoid any bias due to the fluid interpolation onto the larvae, we measured the fluid velocity interpolated to random points in the flow using the same interpolation method. The difference between these two values we call Δ⟨*w*_f_⟩, which is a measure of preferential sampling. When Δ⟨*w*_f_⟩=0, the larvae are not preferentially sampling the flow; when Δ⟨*w*_f_⟩>0, it indicates the larvae oversample upwelling vertical velocities relative to the mean fluid velocity; and when Δ⟨*w*_f_⟩<0, it indicates the opposite.

**Fig. 5.**
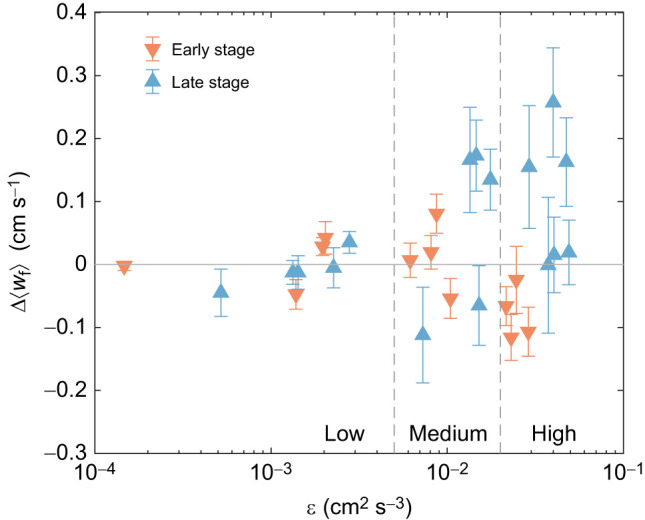
**Average fluid velocity sampled by larvae minus the average fluid velocity sampled by random points in the tank, as a function of the average background turbulence dissipation.** The change in average sampled vertical fluid velocity by the larvae (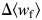) is plotted against dissipation rate (ε) for early- and late-stage larvae. Each data point represents one experimental trial. Positive values indicate enhanced upward transport and negative values indicate reduced transport.

Δ⟨*w*_f_⟩ for each experimental trial is plotted in Fig. 5. For the late-stage larvae, Δ⟨*w*_f_⟩ is close to zero for the low turbulence trials and tends to increase for most of the medium and high turbulence trials, indicating preferential sampling of upwelling flow. The maximum of these values exceeds 2 mm s^−1^, which is more than twice the average vertical swimming speed (measured as relative velocity *w*_r_) for the late-stage larvae in the high turbulence level (where the mean *w*_r_ was measured to be 0.37 mm s^−1^ as reported in Table 1). In contrast, the early-stage larvae do not show strong evidence of preferential sampling, as the values of Δ⟨*w*_f_⟩ in Fig. 5 are all close to zero regardless of the background turbulence level. The values do skew negatively in the high turbulence trials. Therefore, these observations indicate that only the late-stage larvae may be successfully surfing the turbulence in our experiment.

### Larval response to fluid velocity gradient promotes surfing

In the original surfing model, the surfers use information from the full velocity gradient. However, so far we have only considered how the larvae respond to the vorticity. In this section, we further examine the relative importance of the different components of the velocity gradient for both the simulated surfers and the larvae observations.

As previously discussed, the simulated surfers tend to tilt into the vorticity; however, we additionally observed that they also tend to align with the strain axis of maximal extension (see Monthiller, 2022, for more details). We tested whether the larvae in our experiments were similarly aligning with the strain by plotting their horizontal relative velocity as a function of the strain angle and calculating the Pearson correlation coefficient *r* (see Fig. S1 and Table S1). Although we can only make two-dimensional measurements in the lab, we did not observe any strong correlation between the two variables. For example, in the medium and high turbulence levels, |*r*|≤0.1 for both sets of larvae. This suggests that the larvae do not have a strong orientational response to the fluid strain in these experiments. This lack of correlation between the larval relative velocity and the strain angle is unsurprising given how the larvae sense the flow: the larvae have statocysts which can measure rotational motion and translational accelerations, but the larvae sense strain via deformation to their ciliated velum (Budelmann, 1989), which may not be as sensitive to the flow gradients.

**Fig. 6.**
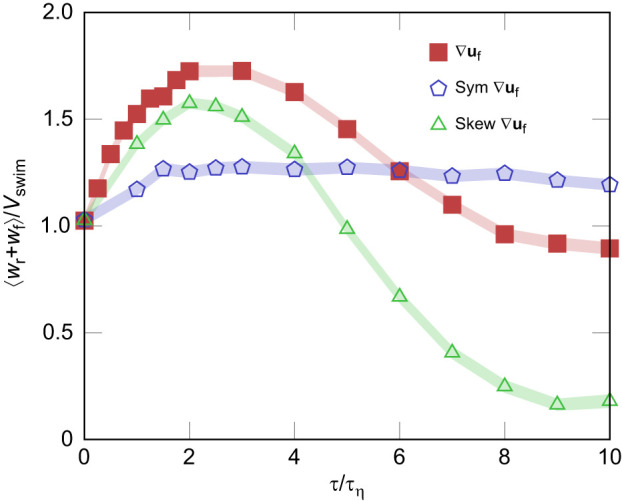
**Vertical migration performance of simulated surfers in turbulence as a function of the surfing time horizon τ for surfers responding to different parts of the full velocity gradient** 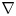
**u_f_.** Surfing using the full velocity gradient, only the symmetric portion (the rate of rotation tensor) and only the skew-symmetric portion (the rate of strain tensor) are plotted. Simulations are evaluated for a swimmer of swimming velocity *V*_swim_=*u*_η_ where *u*_η_ is the Kolmogorov velocity scale. Note that the optimal τ=2τ_η_ corresponds to the peak in the surfing enhancement.

To test the efficiency of surfing by responding only to the vorticity, we conducted additional simulations to test how restricting the sensed information affects the plankton's surfing ability. Instead of using the full velocity gradient in Eqn 5, we tested using only the rate of strain tensor (the symmetric portion of 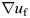) or only the rate of rotation tensor (the skew-symmetric portion of 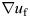). The surfing strategy depends on a free parameter τ, which relates to the time horizon for which the flow velocity gradient 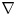
*u*_f_ measured by the plankton can be considered almost constant. The optimal value of this parameter depends on both the swimming speed and the flow, as discussed in Monthiller et al. (2022). The net vertical velocity due to both swimming and preferential sampling normalized by the simulated swim speed as a function of the surfing time horizon is plotted in Fig. 6 for the three different cases. We assessed surfing performance as the degree to which this normalized vertical transport (⟨*w*_r_*+w*_f_⟩/*V*_swim_) exceeds unity, where ⟨*w*_r_*+w*_f_⟩/*V*_swim_=1 denotes when the plankton experience no surfing effect and are transported at their swimming speed *V*_swim_ on average. This shows that while surfers perform best when given the full velocity gradient, those responding solely to the rate of rotation outperform those responding solely to the rate of strain within the optimal surfing range (τ
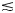
4τ_η_ in this case). This suggests that plankton can still benefit from surfing even if they only respond to fluid vorticity, and that sensing rotation is much more important to surfing than sensing strain. At high τ/τ_η_, surfing performance shows a marked decrease. This is due to the fact that larger time horizons τ underestimate the importance of the unsteadiness in the turbulence velocity gradient; the optimal τ is thought to also be related to the correlation time scale of the turbulence signal experienced by the plankter (Monthiller et al., 2022).

Beyond just vorticity, we can further break down the plankton responses to the individual velocity gradient components in the experiments. If the larvae were indeed actively orienting to increase their upward transport, we would expect them to respond more to the vertical velocity shear d*w*_f_/d*x*, rather than the horizontal velocity shear d*u*_f_/d*z*. This distinction comes from the fact that even though both shear components will rotate the larva, only the former is important to surfing. To elaborate, the surfing strategy optimizes the swimming direction in order to preferentially sample upward fluid velocity. Therefore, it is more important for the surfer to respond to changes in the vertical velocity (e.g. d*w*_f_/d*x* in this case) than to changes in the horizontal velocity (e.g. d*u*_f_/d*z*). Mathematically, this can be further justified by noticing that the surfing strategy in Eqn 5 can be approximated to first order as **p**(*t*)≈
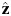
+τ
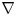
*w*_f_ (Monthiller, 2022). If the larvae were actively rotating in order to stay upright, rather than to surf, we might expect equal responses to both velocity gradients. We assessed the response by again calculating the Pearson correlation coefficient *r* for each shear velocity gradient component, reported in Table 3. In all but one case, we found higher magnitudes of *r* when comparing *u*_r_ with d*w*_f_/d*x* than with d*u*_f_/d*z*. This suggests that the larvae tend to have a stronger orientational response to d*w*_f_/d*x*, which is consistent with the surfing model.

**Table 3.** Pearson correlation coefficient *r* for horizontal relative velocity *u*_r_ correlated against the shear velocity gradients d*u*_f_/d*z* and −d*w*_f_/d*x*, where ω*_y_*=d*u*_f_/d*z*−d*w*_f_/d*x*

Larval stage	Velocity gradient	Turbulence level		
		Low	Medium	High
Early	d*u*_f_/d*z*	*r*=−0.03*	*r*=−0.14	*r*=−0.15
	−d*w*_f_/d*x*	*r*=−0.20	*r*=−0.07	*r*=−0.23
Late	d*u*_f_/d*z*	*r*=−0.26	*r*=−0.14	*r*=−0.25
	−d*w*_f_/d*x*	*r*=−0.36	*r*=−0.34	*r*=−0.32

All *P*<0.001 except for *P=0.015 (two-sided test of *H*_0_: *r*=0).

## DISCUSSION

By carefully observing plankton in turbulence and comparing their swimming behavior with simulations, we have begun to disentangle the coupled effects of their passive and active orientational responses to fluid flow. The veliger larvae of *Crepidula fornicata* are an ideal model organism because of their smooth but varied swimming behaviors, their intermediate Reynolds number, their bottom heaviness and their approximately spherical shape. By analyzing their horizontal relative velocity as a function of the flow around them, we have found evidence that suggests these plankton are capable of actively exploiting turbulent flows to enhance their vertical transport, agreeing with the theoretical ‘surfing’ strategy rather than passive gyrotaxis alone.

We have presented several pieces of evidence that support the active surfing hypothesis. The first is the observed orientational response to the local vorticity. This rotational response must be active as passive reorientation alone cannot result in rotation opposing fluid vorticity, thereby strongly suggesting active behavioral control. For example, at low Reynolds numbers, the torque on a rigid object imposed by the fluid vorticity will always be in the direction of the vorticity, no matter the shape, because the resistance tensor that relates vorticity and viscous torque is positive definite. Thus, in contrast to passive gyrotactic stabilization, the anti-gyrotactic behavior of the larvae we observed must be active because it is in the opposite direction to the vorticity. Previous observations of *C. fornicata* larvae in still water have shown that they do actively vary their swimming direction and speed (DiBenedetto et al., 2021) and can respond to turbulent accelerations by varying their vertical swimming speed (DiBenedetto et al., 2022). These active variations in swimming direction in response to the local flow are consistent with our findings. Finally, while we have described the larvae as approximately spherical in shape, their shells are somewhat flattened and become more asymmetric as they develop. This ontogenetic change in morphology could potentially influence their hydrodynamic response. However, at low Reynolds number, rotation by vorticity is independent of shape, and strain-induced rotation only occurs for elongated shapes (Jeffery, 1922). Therefore, shape changes alone cannot explain the observed responses to vorticity.

The next piece of evidence is that the larvae show a minimal orientational response to the local fluid strain rate. The surfing strategy described by Monthiller et al. (2022) demonstrates optimal surfing occurs when plankton respond to the full velocity gradient. However, simulations show that the rotational component (vorticity) contributes significantly more to successful surfing than the strain component (as shown in Fig. 6). This finding aligns with our observations of larvae primarily responding to vorticity. We note that plankton shape likely influences sensitivity to strain. Elongated plankton naturally tend towards alignment with the extensional rate of strain tensor, enhancing surfing transport passively as has been shown for gyrotactic plankton (Borgnino et al., 2018; Lovecchio et al., 2019). Thus, more elongated plankton may benefit from combining an active response to vorticity with a passive strain alignment, further enhancing their transport. Although our study organism is approximately spherical, future studies involving elongated plankton could provide deeper insights into these combined effects.

The final piece of evidence is the observed preferential sampling of the flow. The late-stage larvae exhibited preferential sampling of upward fluid velocities at medium and high turbulence levels, effectively doubling their net upward velocity relative to their average swimming speed, in close agreement with predictions from surfing simulations. This is consistent with the observation of the late-stage larvae having a stronger anti-vorticity rotational response than the early-stage larvae. The absence of preferential sampling for the early-stage larvae does not necessarily indicate an inability to surf, given the other evidence. The early-stage plankton did typically swim up faster than the late-stage larvae (as was also previously reported in DiBenedetto et al., 2022). Because of the relatively small size of the tank, the larvae can quickly reach and accumulate near the top of the tank. Consequently, our data from observations in the center of the tank may be biased toward slower swimmers or larvae preferentially sampling downwelling regions of the flow. These experimental constraints mean that we cannot definitely assess whether the early-stage larvae were able to effectively surf the flow.

We can compare the late-stage larvae preferential sampling values with the swimming speeds to assess how large the surfing effect would be on their net transport. We reported that the late-stage larvae had an average upward swim speed of less than 0.1 cm s^−1^ in all turbulence levels (although with instantaneous maximum speeds up to 0.33 cm s^−1^) (DiBenedetto et al., 2022). This suggests that surfing can more than double their effective upward velocity by enabling the sampling of higher speed fluid velocities up to and over 0.2 cm s^−1^ on average. Note that the total larval transport in this case is due to the sum of the average swim speed and the average preferential sampling speed. This is similar to the magnitude of increase reported in the optimal surfing simulations (Monthiller et al., 2022), which found that optimal surfing could effectively double the net speed of swimming plankton.

Finally, although we hypothesize that active surfing behavior is causing the larvae to preferentially sample the flow, finite-sized particles can also preferentially sample turbulence due to inertial effects (e.g. Balachandar and Eaton, 2010). However, we argue that the observed preferential sampling is not due to inertial effects, because even in the highest turbulence level, the highest Stokes number *St* of the plankton is of the order 10^−3^. We estimate Stokes number here by taking the ratio *St*=τ_p_/τ_n_, where τ_p_=(ρ_p_−ρ_f_)*d*^2^/18μ_f_ is the Stokes relaxation time scale of a sphere, where ρ_p_ is the density of the larvae and ρ_f_ is the density of seawater. We use the diameter of the larvae and a density of 1.18 g cm^−3^ based on oyster larvae reported in Fuchs et al. (2015b). We find that the early and late stage larvae have τ_p_=0.002 and 0.005 s, respectively. Inertial preferential sampling is typically strongest when Stokes numbers approach unity and is minimal for *St*<<1 (Balachandar and Eaton, 2010), so we do not expect it to play an important role here given the small Stokes number values of *St*≤10^−3^.

In summary, our results demonstrate that relying only on passive reorientation models to describe plankton responses to turbulence is often insufficient, especially for the veliger larvae considered in this study. Although bottom-heavy plankton can passively stabilize their orientation, this does not preclude them from also actively controlling it. In addition to enabling surfing, this active orientational behavior also allows plankton to maintain an upright posture under higher turbulence levels than passive stability alone would permit. Thus, understanding the interplay between active and passive behavioral responses to turbulence is important to fully characterize plankton transport.

## Supplementary Material

10.1242/jexbio.251123_sup1 Supplementary information
