# Supplementary material for: Plankton active response to turbulence enables efficient transport
Source: J Exp Biol. 2025 Dec 19;228(24):jeb251123. doi: 10.1242/jeb.251123 (PMC12752507; doi:10.1242/jeb.251123)
Supplement: Supplementary information [file jexbio-228-251123-s1.pdf]

Larval response to strain angle

Table S1. Pearson correlation coefficient r for horizontal relative velocity u_r correlated against strain angle θ_S as plotted in Fig. S1. All P -values < 0.001 except * where P = 0.01.

Turbulence level	Low		Medium		High	
Early stage	$N = 5036$	r= −0.11	$N = 3995$	r= 0.035*	$N = 6791$	r= −0.059
Late stage	$N = 1243$	r= −0.15	$N = 2102$	r= −0.10	$N = 3675$	r= −0.080

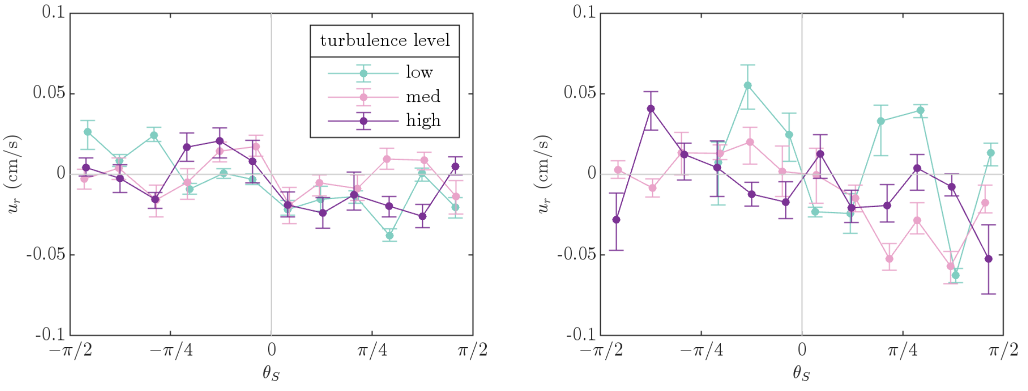

Fig. S1. Larval response to the instantaneous strain angle. Horizontal relative velocity u_r for larvae swimming up is binned by the angle of maximal strain extension in the 2D plane θ_S , and plotted by back-ground turbulence level. (Left) Early-stage larvae and (Right) late-stage larvae data plotted separately. Error bars on bins denote 95% confidence intervals found by bootstrapping the data. Corresponding correlation coefficients reported in Table S1.

Journal of Experimental Biology • Supplementary information
